# A machine learning approach to risk-stratification of gastric cancer based on tumour-infiltrating immune cell profiles

**DOI:** 10.1080/07853890.2025.2489007

**Published:** 2025-04-10

**Authors:** Yanping Hu, Bo Wang, Chao Shi, Pengfei Ren, Chengjuan Zhang, Zhizhong Wang, Jiuzhou Zhao, Jiawen Zheng, Tingjie Wang, Bing Wei, He Zhang, Rentao Yu, Yihang Shen, Jie Ma, Yongjun Guo

**Affiliations:** ^a^ Department of Molecular Pathology, The Affiliated Cancer Hospital of Zhengzhou University & Henan Cancer Hospital, Zhengzhou, China; ^b^ Henan Key Laboratory of Molecular Pathology, Zhengzhou, China; ^c^ Center of Repository, The Affiliated Cancer Hospital of Zhengzhou University & Henan Cancer Hospital, Zhengzhou, China; ^d^ Department of Pathology, The Affiliated Cancer Hospital of Zhengzhou University & Henan Cancer Hospital, Zhengzhou, China; ^e^ Department of Dermatology, The First Affiliated Hospital of Chongqing Medical University, Chongqing, China; ^f^ Central Laboratory, Suzhou Ninth People’s Hospital, Suzhou, China

**Keywords:** Gastric cancer, tumour-infiltrating immune cells, flow cytometry, risk stratification, disease-free survival

## Abstract

**Background:**

Gastric cancer (GC) is a highly heterogeneous disease, and the response of patients to clinical treatment varies substantially. There is no satisfactory strategy for predicting curative effects to date. We aimed to explore a new method for predicting the clinical efficacy of GC treatment based on immune variables detected via flow cytometry.

**Methods:**

We collected 394 tumour tissues from GC patients for flow cytometry analysis and gating analysis of tumour-infiltrating immune cells (TIICs). Unsupervised consensus clusters were generated from the cohort to classify patients into different phenogroups, and their clinical characteristics were examined. The derived model was evaluated via principal component analysis and *t*-distributed stochastic neighbourhood embedding analysis. Kaplan–Meier’s curves were used to determine the prognosis during a 920-day-long median follow-up period (interquartile range: 834–1071 days). Adjusted multivariate Cox regression analysis was used to evaluate the association of clusters with disease-free survival (DFS) and recurrence.

**Results:**

All patients were classified based on their TIIC profiles into the C1 (characterized by low CD45 negative cell, high lymphocyte, high neutrophil and low CD3 + T cell levels), C2 (characterized by high CD8 + CD279+ cell and low CD4+ Th and CD8+ Tc cell numbers) and C3 (characterized by high CD4 + CD25+ and Treg cell levels) phenogroups. Patients from the three clusters had varied pathologies, MMR statuses and TIIC distribution patterns (*p* < .05). Kaplan–Meier’s analysis showed that the prognosis of C3 was inferior compared to C1 and C2 (*p* = .0025). Adjusted Cox proportional hazard models helped us identify that C1 and C2 exhibited a favourable factor of recurrence after surgery, compared to C3. Kaplan–Meier’s analysis showed that C1 and C2 were associated with a better DFS than C3 in some GC patient subgroups.

**Conclusions:**

The machine learning model developed was found to be effective model at predicting the prognosis of patients with GC and their TIIC profiles for risk stratification in clinical settings.

## 1. Introduction

Gastric cancer (GC) is one of the most common cancers worldwide, and it ranks fifth in incidence and mortality among all cancer types [1]. The incidence of GC differs substantially in different regions, which might be attributable to risk factors such as aging, dietary habits and a *Helicobacter pylori* infection. Notably, 43.9% of GC cases and 48.6% of deaths occurred in China [2,3]. In China, GC is the most common malignant tumour of the digestive tract, with a 5-year overall survival rate of 35.1%. The morbidity and mortality rates are the second-highest and third-highest among all malignant cancers, respectively [4]. Hence, there is a compelling need to investigate novel biomarkers that can serve as prognostic indicators for GC, thereby facilitating clinical management of the disease.

Currently, the biomarkers of GC that could provide guidance regarding clinical treatment mainly include the HER2/EREB2 status, microsatellite instability (MSI) or mismatch repair deficiency (MMR) status, programmed death protein ligand-1 (PD-L1) expression, tumour mutation burden (TMB) status and NTRK gene fusion status [5]. However, the current biomarkers cover only a subset of GC cases. For example, HER2 amplification was found only in 13.3% of GC patients [6]. Additionally, due to the heterogeneity of GC, the effectiveness of existing biomarkers varies significantly among individuals, which means they may not provide comprehensive information for all patients. Therefore, there remains a challenge in effectively predicting outcomes due to tumour heterogeneity and the limited predictive ability of current biomarkers. Considering that tumour-infiltrating immune cells (TIICs) directly influence the response of tumours to various therapies, characterizing immune cell profiles might provide a more robust approach to predicting clinical outcomes.

In this study, we aimed to develop a novel method to classify GC patients based on their TIIC profiles, assessed via flow cytometry, thereby improving risk stratification and prognosis prediction in clinical settings.

## 2. Materials and methods

### 2.1. Patient and sample collection

**Figure 1.**
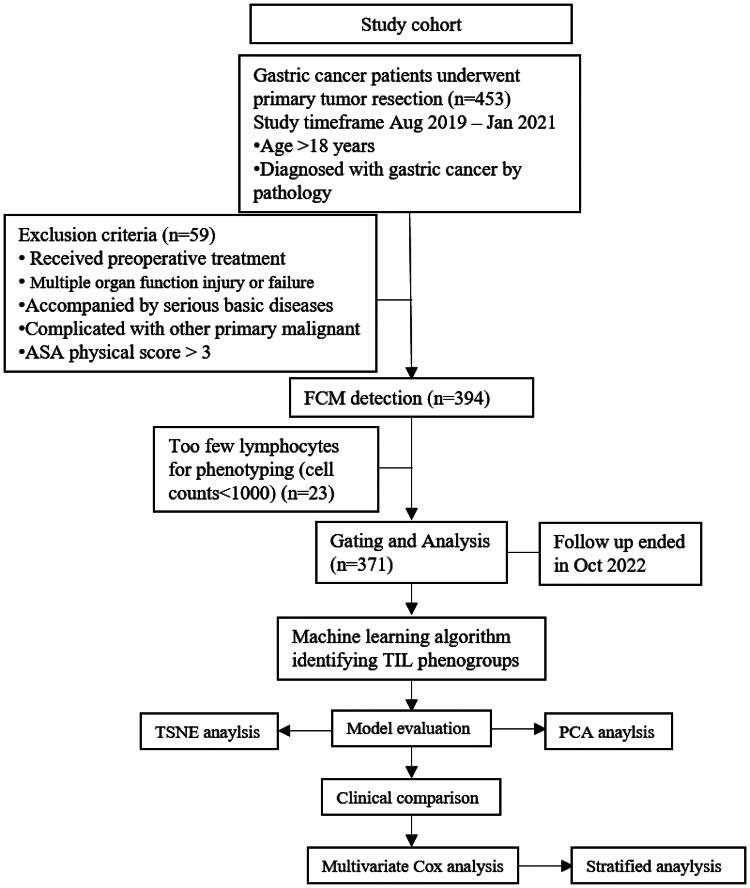
CONSORT-style flow diagram. Study design of FCM analysis of GC patients.

A total of 453 patients with GC were enrolled in our study. All the patients enrolled in this study were from the Henan Province. The patients underwent surgery at the Henan Cancer Hospital (Zhengzhou, China) from August 2019 to January 2021. Tumour tissues were collected and stored in physiological saline. Experienced pathologists and clinicians diagnosed patients in our hospital in accordance with NCCN guidelines [7]. As shown in Figure 1, a total of 371 patients were analysed after filtering out patients using the inclusion and exclusion criteria. The baseline information of patients was queried through the hospital information management system (HIS).

### 2.2. Flow cytometry detection

First, tumour tissue was separated and sheared to prepare a single-cell suspension, filtered using a 200-μm strainer mesh, and washed three times with PBS. Then, 5 μL antibodies and 10^5^ cells were incubated in the dark at room temperature for 15 min. Red cells were disrupted via treatment with 2 mL haemolysin in the dark for 10 min. After washing with PBS three times, 500 μL PBS was added to the solution for testing. Finally, 10,000 lymphocytes were obtained and analysed using the BD Canto system (BD Biosciences, San Jose, CA). The antibody panel used was CD3 FITC (Ref. 349201)/CD 45 PerCP5.5 (Cat. 663499)/CD127 PE (Cat. 557938)/CD8 PEcy7 (Ref. 335822)/CD4 APCcy7 (Cat. 341115)/CD25 APC (Cat. 662525)/CD279 V500 (Cat. 563076). Gates were set and adjusted using a 10-color standard panel that we developed. Initially, human peripheral blood mononuclear cells and isotype controls for each channel were employed to calibrate the voltages. Subsequently, compensation beads were utilized to adjust the compensation settings.

### 2.3. Gating and analysis

**Figure 2.**
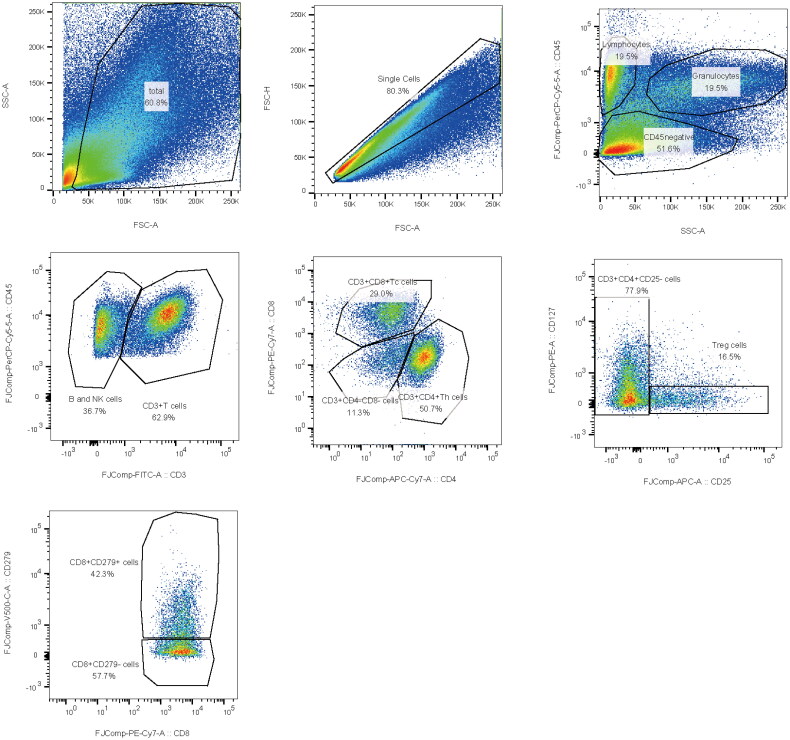
Analysis of the gating strategy of TIIC by flow cytometry.

Kaluza (V2.1) was used to analyse TIICs. Nine TIIC variables, including CD45 negative cells, neutrophils, lymphocytes, CD3+ T cells, CD3 + CD4+ helper T (Th) cells, CD3 + CD8+ cytotoxic T (Tc) cells, CD4 + CD25 + CD127+ regulatory T (Treg) cells, CD4 + CD25+ cells and CD8 + CD279+ cells, were identified for further analysis. First, we removed debris from the FSC/SSC gate and obtained single cells via FSC-A/FSC-H gating. Then, the lymphocytes, neutrophils and CD45 negative cells were gated during CD45/SSC gating. Next, Th and Tc cells were gated during CD3/CD45 gating. Treg cells were observed during CD3/CD4 gating, and CD8 + CD279+ cells were observed during CD3/CD8 gating. CD3-negative cells were identified to be B and NK cells. The gating strategy is shown in Figure 2. FlowJo (V10.8) was used to conduct a *t*-distributed stochastic neighbourhood embedding (*t*-SNE) analysis. Good events were selected using FlowAI, and 1000 events were compressed per FCS file. Subsequently, all the FCS files were concatenated for *t*-SNE analysis. TIIC cells were observed during *t*-SNE1/*t*-SNE2 gating.

### 2.4. Outcomes

Data regarding recurrence events were extracted from the HIS in our hospital, and follow-up was performed until October 2022. Disease-free survival (DFS) was set as the primary endpoint. DFS was defined as the period between the date of surgery and the date of recurrence, metastasis, secondary primary tumour or death. Patients without recurrence until the date of the last follow-up were counted. Tumours were evaluated according to RECIST (version 1.1) guidelines.

### 2.5. Unsupervised machine learning clustering analysis

Clustering was performed using unsupervised machine learning clustering analysis. Values of the nine TIIC variables were *Z*-score transformed before clustering. The ‘ConsensusClusterPlus’ package in R [8] was used to determine the optimal cluster K (Supplementary Figure S1). Briefly, this algorithm initiates by repeatedly subsampling a random subset of features from the dataset and partitioning the data into k clusters using methods such as agglomerative hierarchical clustering, k-means or a custom approach. This process is iterated multiple times to ensure stability, after which pairwise consensus values – representing how frequently pairs of data points are grouped together across iterations – are aggregated into a consensus matrix for each candidate k. For each k, the consensus matrix is transformed into a distance metric (1 − consensus), and agglomerative hierarchical clustering is applied to this matrix to generate a dendrogram, which is then pruned to yield k final consensus clusters. Clustering performance was evaluated using the ‘fpc’ package in R to ensure robustness and interpretability. Internal validation metrics, including the Silhouette coefficient, Calinski–Harabasz index and Dunn index, were computed via the cluster.stats function to quantify intra-cluster cohesion and inter-cluster separation. Cluster stability was assessed through a bootstrap resampling approach (50 iterations) using clusterboot, which measured the consistency of partitions via Jaccard similarity. Finally, the CLARA algorithm, wardD method and Euclidean distance were applied for consensus clustering (Supplementary Table S1). The results of the clustering analysis are shown in Supplementary Figure S2a.

Principal component analysis (PCA) was performed to assess the discriminative performance of the unsupervised algorithm in TIIC signatures and used as a dimensionality reduction technique to describe overall changes. The first three principal components (PCs), which accounted for more than 80% of the variance, were selected for further analysis. Finally, all patients were mapped into a 2D coordinate system based on the three PCs (Supplementary Figure 2B). The *t*-SNE method was also used to visualize low-dimension characteristics. A relatively clear boundary was observed when the dimension equalled 3, suggesting significant differences among these patients (Supplementary Figure 2C).

### 2.6. Statistical analysis

Continuous and categorical variables were summarized as medians (interquartile ranges) and frequencies (percentages). The differences among the three clusters were compared using the Kruskal–Wallis or Chi-squared test. The log-rank test was used to compare Kaplan–Meier’s curves for DFS among the three clusters. Multivariate Cox proportional hazard regression was used to explore the association between the clusters and recurrence. All statistical analyses were conducted with R version 4.22 (R Foundation for Statistical Computing, Vienna, Austria). A two-tailed *p* value of ≤.05 was considered to be statistically significant.

## 3. Results

### 3.1. Unsupervised clustering-based TIIC phenogroups and their characteristics

**Figure 3.**
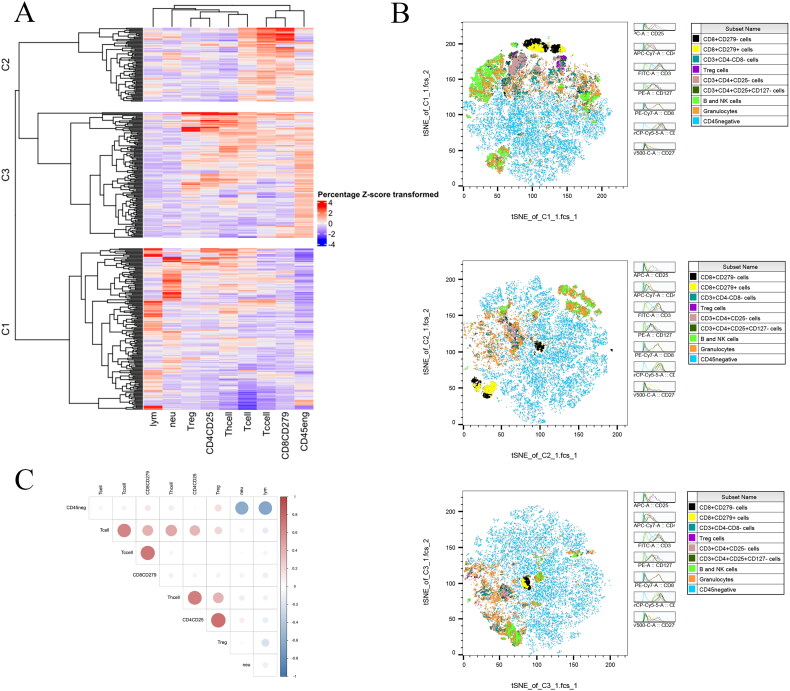
TIIC profiles of GC patients clustered using machine learning techniques. (A) Heatmap of the three phenogroups. (B) *t*-SNE analysis of TIIC profiles using FlowJo. (C) Intercorrelation among different TIIC variables.

The nine TIIC variables were classified into three clusters, i.e. cluster 1 (C1), cluster 2 (C2) and cluster 3 (C3), using the unsupervised machine learning algorithm. C1 was characterized by low CD45 negative cell, high lymphocyte, high neutrophil and low T cell numbers. C2 was characterized by high CD8CD279 cell, low Th cell and high Tc cell numbers. Conversely, C3 was characterized by high CD4CD25 cell and high Treg cell numbers. The visual signatures of the three clusters are shown in Figure 3(a). Moreover, *t*-SNE results showed that the three groups exhibited different phenotypes, as shown in Figure 3(b). In addition, we conducted a Pearson correlation analysis to assess the nine variables. There were strong positive correlations between T cell and Tc cell, Tc cell and CD8 + CD279+ cell, Th cell and CD4 + CD25+ cell, CD4 + CD25+ cell and Treg cell; meanwhile, a strong negative correlations were found between neutrophils and CD45 negative cell, lymphocytes and CD45 negative cell (Figure 3(c)). In addition, there were significant differences in the proportions of the nine TIIC variables among the three clusters (*p* < .05), as shown in Table 1.

**Table 1.** Relative quantitation of subtype of immune cells by clusters.

	Cluster 1 (*N* = 166)	Cluster 2 (*N* = 76)	Cluster 3 (*N* = 129)	*p*
Neutrophils	21 (11, 34)	15 (7, 23)	9 (6, 14)	<.001
CD45 negative cell	39 (28, 48)	54 (48, 61)	67 (61, 72)	<.001
Lymphocytes	20 (11, 35)	15 (7, 22)	7 (4, 11)	<.001
T cell	61 (49, 72)	75 (70, 85)	72 (59, 80)	<.001
Th cell	30 (21, 37)	22 (16, 26)	35 (24, 42)	<.001
Tc cell	23 (16, 30)	50 (43, 58)	28 (23, 37)	<.001
CD4 + CD25+ cell	6 (3, 10)	5 (3, 8)	10 (6, 16)	<.001
Treg	1.9 (0.8, 3.7)	1.2 (0.6, 3.0)	3.5 (1.6, 6.4)	<.001
CD8 + CD279+ cell	6 (3, 10)	21 (13, 34)	13 (6, 19)	<.001

Cluster 1: CD45 negative cell low, lymphocytes high, neutrophils high and T cell low. Cluster 2: CD8 + CD279+ cell high, Th cell low and Tc cell low. Cluster 3: CD4 + CD25+ cell high and Treg high.

Data were described as median (interquartile range). Neutrophils, CD45 negative cell and lymphocytes were the ratio to all acquired cell. T cell, Th cell, Tc cell, CD4 + CD25+ cell, Treg and CD8 + CD279+ cells were the ratio to lymphocytes.

### 3.2. Baseline data

The baseline characteristics of the 371 GC patients that fulfilled the inclusion criteria are shown in Table 2. The average age of the entire cohort, which included 82 males and 289 females, was 62.57 (55–69) years. The patients were divided into three groups based on the machine learning-based clustering of TIIC data. There was no significant difference in most of the baseline data, including the sex, age, differentiation, primary site, nerve invasion, venous invasion, Lauren classification, TNM staging, HER2 status, Ki67 status, PD-L1 status and adjuvant therapy in the three groups. However, there were statistical differences in the pathological types and MMR statuses in the three clusters. Cluster 1 had the lowest proportion of adenocarcinomas and the highest proportion of signet-ring cell carcinomas. Cluster 2 had the highest proportion of adenocarcinomas and the lowest proportion of signet-ring cell carcinomas (*p* < .05). The MSI ratio in the three groups was the highest for the C2 cluster, followed by that for C3 and C1 (*p* < .05).

**Table 2.** Baseline characteristics of patients categorized by clusters.

	Cluster 1 (*N* = 166)	Cluster 2 (*N* = 76)	Cluster 3 (*N* = 129)	*p*
**Sex**				.18
Male	44 (26.51%)	14 (18.42%)	24 (18.60%)	
Female	122 (73.496%)	62 (81.58%)	105 (81.40%)	
Age	64 (55, 69)	64 (59, 69)	65 (56, 70)	.34
Pathological types				.04
Adenocarcinoma	125 (75.76%)	68 (89.47%)	113 (87.60%)	
Signet-ring cell carcinoma	31 (18.79%)	7 (9.21%)	12 (9.30%)	
Others	9 (5.45%)	1 (1.32%)	4 (3.10%)	
Unknown	1	0	0	
Differentiation				.62
Poor	122 (82.43%)	57 (78.08%)	97 (78.23%)	
Well to moderate	26 (17.57%)	16 (21.92%)	27 (21.77%)	
Unknown	18	3	5	
Site				.11
Body	46 (28.75%)	31 (41.33%)	42 (33.07%)	
Proximal	69 (43.13%)	22 (29.33%)	58 (45.67%)	
Remote	45 (28.13%)	22 (29.33%)	27 (21.26%)	
Unknown	6	1	2	
Nerve invasion	111 (72.08%)	53 (71.62%)	81 (64.80%)	.38
Unknown	12	2	4	
Venous invasion	112 (72.73%)	56 (75.68%)	96 (76.80%)	.72
Unknown	12	2	4	
Lauren classification				.1
Diffuse	47 (43.52%)	17 (33.33%)	22 (26.19%)	
Intestine	23 (21.30%)	16 (31.37%)	30 (35.71%)	
Mixed	38 (34.94%)	18 (35.29%)	32 (38.10%)	
Unknown	58	25	45	
TNM				.88
I–II	61 (38.61%)	27 (36.00%)	46 (35.94%)	
III–IV	97 (61.39%)	48 (64.00%)	82 (64.06%)	
Unknown	8	1	1	
Adjuvant therapy				.94
Chemo	104 (88.89%)	46 (90.20%)	77 (89.53%)	
Chemo + targeting	6 (5.13%)	2 (3.92%)	4 (4.65%)	
Chemo + ICI	4 (3.42%)	2 (3.92%)	5 (5.81%)	
Targeting	2 (1.71%)	0 (0%)	0 (0%)	
Chemo + targeting + ICI	0 (0%)	0 (0%)	1 (1.16%)	
ICI + targeting	1 (0.85%)	0 (0%)	0 (0%)	
Unknown	49	25	43	
MMR				.02
MSI	2 (1.54%)	7 (10.29%)	6 (5.36%)	
MSS	128 (98.46%)	61 (89.71%)	106 (94.64%)	
Unknown	36	8	17	
Her2 expression				.07
Negative	67 (74.44%)	39 (79.59%)	62 (69.66%)	
Positive	23 (25.56%)	10 (20.41%)	27 (30.34%)	
Unknown	76	27	40	
Ki67 positive	58 (34.94%)	27 (35.53%)	50 (38.76%)	.79
PDL1 positive	35 (21.08%)	22 (28.95%)	25 (19.38%)	.26
Adjuvant therapy	123 (74.10%)	52 (68.42%)	92 (71.32%)	.65

ICI: immune checkpoint inhibitors.

### 3.3. Disease-free survival in different clusters

**Figure 4.**
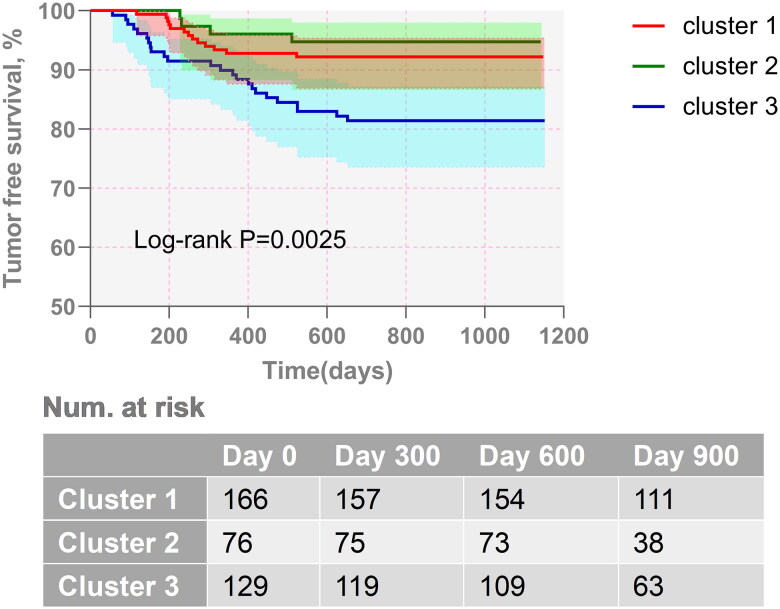
Kaplan–Meier’s curves among different phenogroups. The upper figure shows the survival curves by different clusters, and the lower figure shows the risk table.

During follow-up, 41 events were registered among 371 patients. The Kaplan–Meier survival curve indicated that the DFS of C3 was significantly lower than that in C1 and C2 (log-rank *p* = .0025, Figure 4). To investigate the exact role of the TIIC profile in determining the prognosis of GC patients further, we generated multivariate Cox regression models adjusted by clinical factors. The Cox proportional hazard models identified the TIIC profile to be a risk factor for DFS, even after adjusting for age, sex, primary site, pathological type, differentiation, Lauren classification, TNM staging, MMR status, HER2 status, Ki67 status, PD-L1 status and adjuvant therapy (HR = 0.36–0.43, *p* < .05 for all, Table 3). Meanwhile, the 1-year DFS and 3-year DFS values of the C1 and C2 clusters were better than those of C3, as shown in Table 4.

**Table 3.** COX regression analysis of recurrence after surgery in different multivariate models.

	Cluster 1 (*N* = 166)			Cluster 2 (*N* = 76)		
	HR	95%CI	*p*	HR	95%CI	*p*
Univariate	0.40	0.20–0.78	.008	0.26	0.09–0.76	.013
Model 1	0.40	0.20–0.80	.009	0.24	0.08–0.68	.008
Model 2	0.43	0.21–0.86	.017	0.23	0.08–0.68	.007
Model 3	0.37	0.19–0.76	.006	0.23	0.08–0.67	.007
Model 4	0.36	0.18–0.74	.005	0.23	0.08–0.67	.007
Model 5	0.39	0.20–0.78	.008	0.25	0.09–0.71	.010

Model 1: adjusted by age, gender and primary site. Model 2: model 1 + pathological type and differentiation. Model 3: model 1 + Lauren classification + TNM. Model 4: model 1 + MMR + Her2 + Ki67 + PDL1. Model 5: model 1 + adjuvant therapy.

Cluster 3 as reference.

**Table 4.** One- and three-year disease free survival rate after surgery by clusters.

	1-Year DFS	3-Year DFS	HR	95%CI	*p*
Cluster 1	92.8%	92.2%	0.40	0.20–0.78	.008
Cluster 2	96.1%	94.7%	0.26	0.09–0.76	.013
Cluster 3	89.2%	81.4%	Ref.	Ref.	Ref.

### 3.4. The effect of TIIC profiles on DFS in different GC subgroups

**Figure 5.**
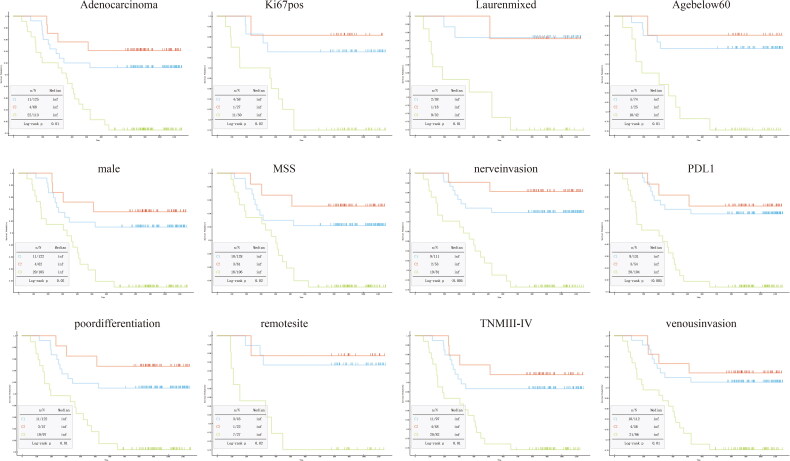
Kaplan–Meier’s curves of TIIC profiles of patients exhibiting DFS in subgroups of patients with adenocarcinomas (A); patients undergoing adjuvant therapy (B); those who were HER2 positive (C), ki67 positive (D) and PD-L1 negative patients (E); and those with mixed Lauren types (F), MSS status (G), nerve invasion (H), poor differentiation (I), remote tumour sites (J), TNM stages III–IV (K) and venous invasion (L).

Kaplan–Meier’s analysis showed that C1 and C2 were associated with a better DFS than C3 in some subgroups of GC patients, including those with adenocarcinomas, ki67 positive, PD-L1 negative, and those with mixed Lauren types, MSS status, nerve invasion, poor differentiation, remote tumour sites, TNM stages III–IV and venous invasion (Figure 5).

## 4. Discussion

Gastric cancer is characterized by highly heterogeneous tumours whose biological behaviour is affected by the complicated gene regulatory network of the cells and immune microenvironment [9]. Recent studies have emphasized that immune cell subsets, especially T cells and CAFs, play a critical role in shaping the immune landscape and prognosis of GC [9,10]. Due to the growing interest in immunotherapy, numerous studies have focused on the immunophenotyping of GC microenvironment. Chen et al. [4] established an immune phenotyping technique involving CD4 + FoxP3 − PD-L1+, CD8 + PD-1 − LAG3− and CD68 + STING+ cells for predicting patient survival and GC patient response to anti-PD-1/PD-L1 immunotherapy [11]. Li et al. identified iCAFs and eCAFs in GC through single-cell RNA sequencing, revealing their pro-invasive activities and their role in mobilizing surrounding immune cells, thus contributing to a tumour-promoting microenvironment [12]. The primary distinctions between our study and previous research lie in the methodologies and target immune cells selected for analysis. Specifically, unlike traditional clustering methods such as K-means, this study utilized the CLARA algorithm, which is robust to outliers and more computationally efficient for analysing large and dispersed datasets. Additionally, *t*-SNE algorithm was employed for visualization, effectively preserving local data structure and clearly demonstrating differences among identified immune subtypes.

Currently, the immune microenvironment is mainly evaluated using multiple immunohistochemistry (mIHC) and next-generation sequencing (NGS) methods [13,14]. The mIHC method is intuitive and effective for evaluating spatial locations, but the clustering process is not optimal. NGS is a more objective method; however, it cannot be used for protein detection. Flow cytometry can detect more refined and specific immune cell subsets, but cannot identify the spatial distribution. Here, we developed a novel approach for the risk stratification of the immune microenvironment based on flow cytometry detection methods. The new method for gastric tumour classification based on the immune microenvironment is expected to become an important basis for determining prognoses and identifying potential therapeutic beneficiaries.

In our previous study, we established the flow cytometry methodology for detecting the contents of lymphocyte subsets in individuals with colorectal cancer (CRC). We found that CD8 + CD279+ cells were superior prognostic markers of CRC [15]. However, according to our unpublished data, the results of an ROC analysis to evaluate the role of CD8 + CD279+ cells in CRC patients showed that the area under the curve value was 0.561. This indicated that the specificity of CD8 + CD279+ cells in DFS was not significant in this model of CRC. It showed that a single indicator could not be used for optimal prognosis prediction. Therefore, we combined multiple indicators to establish a new risk stratification model that could provide better guidance regarding the clinical treatment of GC in this study.

In this study, we classified GC patients into three groups based on their TIIC characteristics, among which high CD8CD279, low Th cell and high Tc cell numbers characterized the C2 cluster. These results are consistent with our previous study, which showed that CD8 + CD279+ cells had a superior effect on CRC prognosis [16]. Similarly, numerous studies have demonstrated the positive effects of CD8 + CD279+ cells [17–19]. Tc cells play a role in cellular immunity and kill tumour cells directly, while high Tc cell numbers usually indicate a better prognosis. In our study, a higher proportion of Tc cells were observed in C2, which is consistent with the results of previous studies [20–23]. The worst prognosis in our model was obtained for the C3 cluster, which was characterized by a high proportion of Treg cells. The Treg cell is an important immunosuppressive cell, whose role in tumours is currently controversial. It is generally considered to be associated with an inferior prognosis in breast, gastric and CRC patients [24–26], and a superior prognosis in ovarian cancer patients [27], which may be attributable to the heterogeneity of Treg cells [28]. CD127 is also a specific surface marker of Treg-like FOXP3, which is suitable for large-scale sample detection [29]. Hence, we used CD4 + CD25 + CD127dim + instead of FOXP3 for Treg cell gating. These findings underscore the critical role of immune profiles as prognostic indicators, potentially guiding individualized treatment strategies in clinical practice.

Certain shortcomings are associated with our study. First, the detected subgroups were not fine enough. In addition, the FCM method has inherent defects related to the determination of the spatial distribution of cells, which represents an important factor affecting the tumour immune microenvironment.

The findings from the immune classification of GC based on TIIC profiles have potential clinical applications. Specifically, they suggest biomarkers that may be used for GC prognosis as well as potential targets for clinical developing interventions. While our study introduces an effective model for prognostication in GC patients, further research employing more refined clustering is warranted. Future investigations should prioritize validating TIIC profiles for risk stratification and establishing cut-off values for various parameters to enhance the clinical applicability of our findings.

## 5. Conclusions

Collectively, the findings from the immune classification of GC based on TIIC profiles have promising clinical applications. Specifically, the identified immune subsets suggest biomarkers that could inform prognosis predictions and guide personalized therapeutic interventions for GC.

## Supplementary Material

Supplemental Material

## Data Availability

The raw data are available from the corresponding author on reasonable request.
