# Supplementary material for: A machine learning approach to risk-stratification of gastric cancer based on tumour-infiltrating immune cell profiles
Source: Ann Med. 2025 Apr 10;57(1):2489007. doi: 10.1080/07853890.2025.2489007 (PMC11986862; doi:10.1080/07853890.2025.2489007)
Supplement: Supplemental Material [file IANN_A_2489007_SM5847.zip › Suppl/Suppl_Fig Caption.docx]

**Supplementary Figure S1**. Analysis of optimal cluster K. (A) Heatmap of the consensus matrix between 2 and 5. (B) Empirical cumulative distribution function plot when k values = 2 to 5; (C) Sum of the squared errors of k values ranging from 2 to 10.

**Supplementary Figure S2**. Evaluation of TIIC signatures using the model via unsupervised machine learning clustering analysis. (A) Silhouette plot of different clusters. (B) Cluster plot of PCA results. (C) Visualization of three-dimensional t-SNE transfer.
