# Supplementary figures and images for: A machine learning approach to risk-stratification of gastric cancer based on tumour-infiltrating immune cell profiles
Source: Ann Med. 2025 Apr 10;57(1):2489007. doi: 10.1080/07853890.2025.2489007 (PMC11986862; doi:10.1080/07853890.2025.2489007)

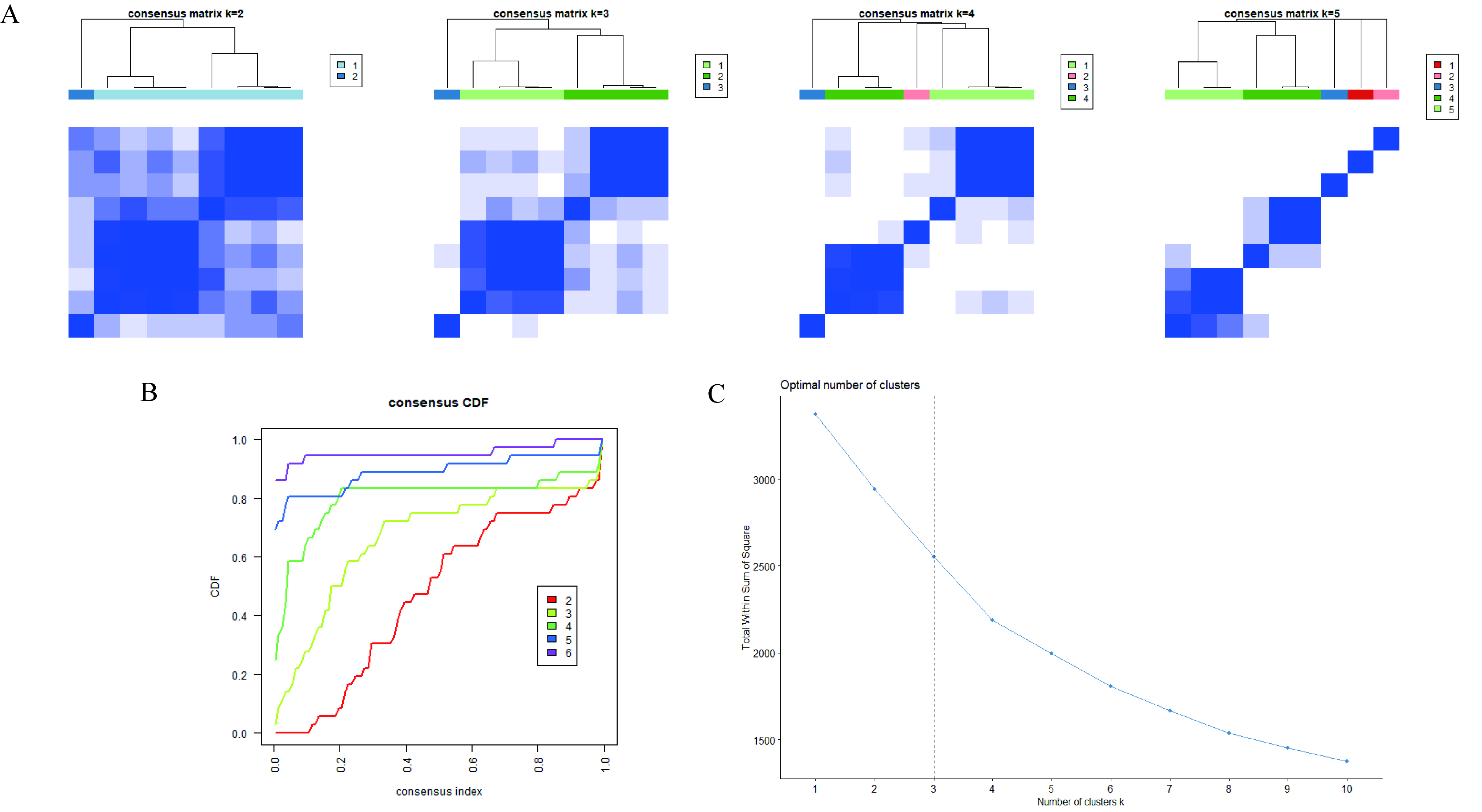

Supplement: Supplemental Material [file IANN_A_2489007_SM5847.zip › Suppl/FigS1 (2).tif]

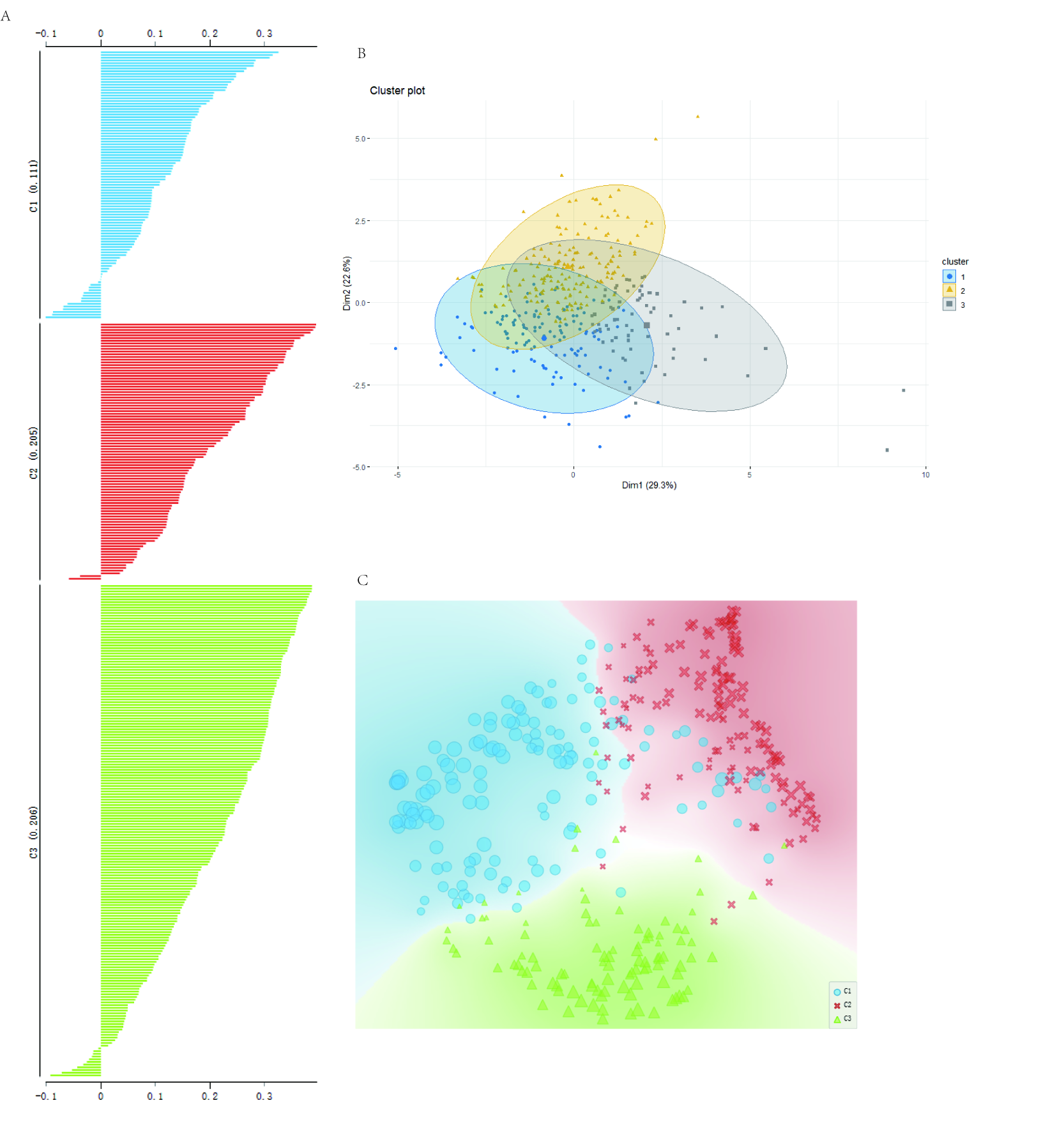

Supplement: Supplemental Material [file IANN_A_2489007_SM5847.zip › Suppl/FigS2 (2).tif]
